# Identification of a genetic variant underlying familial cases of recurrent benign paroxysmal positional vertigo

**DOI:** 10.1371/journal.pone.0251386

**Published:** 2021-05-06

**Authors:** Yinfang Xu, Yan Zhang, Ivan A. Lopez, Jacey Hilbers, Anthony J. Griswold, Akira Ishiyama, Susan Blanton, Xue Zhong Liu, Yunxia Wang Lundberg

**Affiliations:** 1 Vestibular Genetics Laboratory, Boys Town National Research Hospital, Omaha, Nebraska, United States of America; 2 Department of Head and Neck Surgery, “David Geffen” School of Medicine at The University of California at Los Angeles, Los Angeles, California, United States of America; 3 Department of Human Genetics and John P. Hussman Institute for Human Genomics, Dr. John T. Macdonald Foundation, University of Miami Miller School of Medicine, Miami, Florida, United States of America; 4 Department of Otolaryngology, The University of Miami Miller School of Medicine, Miami, Florida, United States of America; CNR, ITALY

## Abstract

Benign paroxysmal positional vertigo (BPPV) is the most common cause of vertigo in humans, yet the molecular etiology is currently unknown. Evidence suggests that genetic factors may play an important role in some cases of idiopathic BPPV, particularly in familial cases, but the responsible genetic variants have not been identified. In this study, we performed whole exome sequencing [including untranslated regions (UTRs)] of 12 families and Sanger sequencing of additional 30 families with recurrent BPPV in Caucasians from the United States (US) Midwest region, to identify the genetic variants responsible for heightened susceptibility to BPPV. Fifty non-BPPV families were included as controls. *In silico* and experimental analyses of candidate variants show that an insertion variant rs113784532 (frameshift causing truncation) in the neural cadherin gene *PCDHGA10* (protocadherin-gamma A10) is an exceedingly strong candidate (p = 1.80x10^-4^ vs. sample controls; p = 5.85x10^-19^ vs. ExAC data; p = 4.9x10^-3^ vs. NHLBI exome data). The mutant protein forms large aggregates in BPPV samples even at young ages, and affected subjects carrying this variant have an earlier onset of the condition than those without [average 44.0±14.0 (n = 16) versus 54.4±16.1 (n = 36) years old, p = 0.054]. In both human and mouse inner ear tissues, PCDHGA10 is expressed in ganglia, hair cells and vestibular transitional epithelia. Fluorescent RNA *in situ* hybridization using mouse inner ear tissues shows that expression increases with age. In summary, our data show that a variant in the *PCDHGA10* gene may be involved in causing or aggravating some familial cases of recurrent idiopathic BPPV.

## Introduction

Benign paroxysmal positional vertigo (BPPV) is a vestibular condition believed to be caused by otoconia dislocation from the utricle (which senses linear head motion) to the semicircular canals (which detect rotational movement). With a lifetime prevalence of 10% [1], BPPV is the most common cause of vertigo in humans. The prevalence drastically increases at middle age and older [2–5], and an additional ~9% of elderly persons have undiagnosed BPPV [6]. Triggered by rapid changes of head position, BPPV episodes can be intense, and often cause nausea and vomiting. Therefore, the word “benign” in the term BPPV is only in the sense that the condition is not life-threatening; the vertigo itself is not benign at all. In fact, BPPV can be debilitating and disruptive to daily living [7,8], and is much more incapacitating and persistent in the elderly (e.g. falls and the inability to get up on their own or even death) [9,10].

Although BPPV is largely (~70%) treatable with maneuvers to reposition the dislocated otoconia in the utricle, there is a high recurrence rate of about 30% within the first year and 50% after 5 years [11–13]. While one third of BPPV cases in young people can be attributed to head trauma/injury, idiopathic BPPV cases are much more common in middle-aged and older people, which also tend to be recurrent [4,14,15]. Currently, no medication is available to treat or prevent BPPV. Therefore, the development of effective treatment or prevention for recurrent cases is needed and is dependent on research on the molecular etiology of BPPV.

Emerging evidence suggests that BPPV is a disorder with heterogeneous environmental [14,16–19] and genetic causes [4,20,21]. A previous study in our laboratory shows familial predisposition in BPPV occurrence [4]. Indeed, a genetic analysis of a three-generation family in which multiple family members developed BPPV mapped the trait to chromosome 15 [20]. Another genetic study [21] mapped recurrent vertigo to chromosome 22q12. The paper by Lee et al. [21] only referred to the symptom as recurrent vertigo and did not say whether BPPV diagnostic criteria were used. Many patients in this study also had migraine, so it is possible that not all of them had BPPV *per se*. The above reports show that familial cases of BPPV have an autosomal dominant inheritance with reduced penetrance. A recent study [22] performed targeted sequencing of the following 3 candidate genes in 726 BPPV patients (presumably unrelated) and 502 normal controls: vitamin D receptor gene, and two genes (*LOXL1* and *LOXL1*-*AS1*) within the critical interval on chromosome 15 previously mapped by Gizzi et al., 2015 [20]. This candidate gene approach identified an intronic variant rs1078967 in the gene *LOXL1* which may be associated with BPPV, but the functional importance of this variant is unknown. In this report, we used a family-based case-control strategy and performed whole exome sequencing in familial cases of BPPV to search for candidate variants that may be responsible for heightened susceptibility to recurrent BPPV.

## Materials and methods

### Human subjects

This study was approved by the Institutional Review Board (IRB) at Boys Town National Research Hospital (BTNRH) (approval number 15-01-F) in accordance with institutional, federal and international guidelines.

Participants were recruited through Medical Records at BTNRH, flyers, web postings, and participants themselves. Familial cases were identified either through medical records, or through participants. The affected relatives were confirmed to be diagnosed with BPPV by an otolaryngologist or audiologist at BTNRH or elsewhere.

The study goal, procedure, risks and benefits were explained to individuals who responded to the study invitation before signing the informed consent and HIPAA (Health Insurance Portability and Accountability Act) authorization forms. Subjects were then asked to collect their saliva samples using the Oragene^™^ Self-Collection Kits (Cat# OGR-500, DNA Genotek Inc., Ottawa, ON, Canada) and complete a detailed questionnaire through Survey Monkey (www.surveymonkey.com) or on paper. The comprehensive questionnaire asked about demographic information (name, sex, age, weight, height, race and whether live in rural or urban area), BPPV related information (timeframe and location of diagnosis, method of treatment, recurrence, family history, season of onset, incidents or conditions prior to BPPV onset and comorbidities accompanying BPPV symptoms), medical history, current and previous medications, dietary habits and supplementation, family history of hearing loss/deafness and other hearing problems, whether and when participants had experienced natural or surgical menopause (hysterectomy/oophorectomy) in women and prostate removal surgery in men, use of estrogen and other hormone therapy, and whether the above conditions/situations coincided with or preceded the onset of BPPV.

Inclusion criteria for the enrollment of familial BPPV cases were: (1) at least two blood relatives (live or deceased) in the family had been clinically diagnosed with BPPV, and at least one relative had recurrent BPPV; (2) unaffected blood relatives were approximately 20 years older than the onset age of the affected family member and never had BPPV (the older age requirement is to minimize the likelihood that the individual will develop BPPV later in life), or were 60 years or older; (3) the subject signed informed consent and HIPAA authorization forms, and completed the questionnaire. Selection of families for whole exome sequencing (WES) was based on the availability of multiple samples at the time, and no other major illness as described in exclusion criteria below.

Inclusion criteria for the enrollment of independent controls were: (1) subjects were in a similar age range as the unaffected controls in the familial cases (the mean age of the independent controls was 62); (2) subjects and blood relatives never had BPPV; (3) subjects and blood relatives did not have hearing impairments or other chronic hearing problems; (4) subjects signed informed consent and HIPAA authorization forms, and completed the questionnaire.

Inclusion criteria for the enrollment of non-BPPV families were similar to those for the independent controls. These controls were used to obtain the sample allele frequency of identified variants in non-BPPV families, because the BPPV status in available public databases is not known.

Exclusion criteria were head trauma, serious infection (especially toxoplasmosis, rubella, cytomegalovirus, Herpes simplex virus, meningitis), aminoglycoside exposure, significant low birth weight (<4 lb, if known), inner ear structural abnormalities (if known), hyperbilirubinemia, and autoimmune disease (diabetes, autoimmune thyroid diseases, multiple sclerosis, myasthenia gravis). If affected family members did not share the same comorbidity, then the condition was not used as an exclusion criterion.

### Genomic DNA preparation

Saliva samples were collected from the participants using Oragene^™^ Self-Collection Kits (Cat# OGR500), and genomic DNA (gDNA) was extracted using the Oragene prepIT-L2P kit (Cat# PT-L2P-5, DNA Genotek Inc.) following the manufacturer’s protocol. The quality and concentration of gDNA was evaluated by agarose gel electrophoresis and NanoDrop One^C^ spectrophotometer (Thermo Fisher Scientific, Wilmington, DE, USA).

### Whole exome sequencing (WES) and bioinformatic analysis

WES+UTR sequencing and initial bioinformatic analysis were performed by Otogenetics Corporation (Atlanta, GA, USA). Briefly, high molecular weight gDNA with good integrity and good optical density ratio at 260/280 (~1.8) was fragmented using a Bioruptor sonicator (Diagenode, Inc., Denville, NJ, USA), and tested for size distribution and concentration using an Agilent Tapestation 2200 and Nanodrop. Illumina libraries were then made from qualified fragmented gDNA using SPRIworks HT Reagent Kit (Cat# B06938, Beckman Coulter, Inc. Indianapolis, IN, USA) and the resulting libraries were subjected to exome enrichment using SureSelect^XT^ Human All Exon version 5-UTRs (Cat# 5190–6215, Agilent Technologies, Wilmington, DE, USA) following manufacturer’s instructions. Enriched libraries were tested by an Agilent Bioanalyzer 2100 and sequenced on an Illumina HiSeq2500 (Illumina, San Diego, CA, USA), which generated 106-bp paired-end reads with an average of 63x coverage. The quality of raw data was analyzed with FASTQC (Babraham Institute, Cambridge, UK).

After removing adapter sequences and low quality reads (e.g. too many Ns or low quality bases), the clean data were mapped against the human reference genome (GRCh37/hg19) using BWA algorithm with default parameters [23]. Duplicate reads were removed using Picard (http://picard.sourceforge.net). The Genome Analysis Tool Kit (GATK-Lite) toolkit v3.8 [24] module IndelRealigner and BaseRecalibrator were used to preprocess the alignments. During base quality recalibration, dbSNP141 variants were used as known sites, according to GATK Best Practices recommendations [25]. Target-capture efficiency metrics were determined using Target region coverage calculator Version 0.0.1. The realigned and recalibrated BAM file was used as an input to UnifiedGenotyper module from the GATK-lite toolkit. Variant calls were restricted to the target regions (Agilent SureSelectXT Human All Exon V5+UTR). Variants were annotated with SnpEff v4.1 and SnpSift 4.0 [26] using open-source databases (e.g. dbSNP, ClinVar, ExAc, 1000 Genomes, etc.). Finally, sorted mappings index and mapping summaries were obtained by using samtools v1.8 [27].

After removing low impact variants according to SnpEff/SnpSift, those meeting the following criteria were prioritized for Sanger sequencing of additional samples: (1) variants are shared by affected but not by unaffected family members; (2) variants have a minor allele frequency (MAF) of lower than 0.05, or a CADD (combined annotation dependent depletion) score above 9; (3) variants are shared by 2 or more families, known to be expressed in the inner ear according to Gene Expression Omnibus (GEO) or Pubmed, or known to affect balance/hearing according to Online Mendelian Inheritance in Man (OMIM) or Pubmed. Sanger sequencing of the candidate variants was also performed on the WES samples for confirmation. Because BPPV has a lifetime prevalence of 10% [1], and not all cases have a genetic etiology, we used the criteria of MAF<0.05 assuming genetic heterogeneity (i.e. several genes are involved). Neither criterion for the MAF or CADD score was stringent, so as to minimize false negatives at this initial screening stage.

### Sanger sequencing of human saliva samples

Sanger sequencing was performed to confirm the variants identified by WES, and to determine whether the variants in potentially causative genes co-segregated with the disease phenotype in additional BPPV families and independent controls whose samples did not undergo WES. Forward and reverse primers (listed in S1 Table) were used to amplify the regions containing the candidate variations. The polymerase chain reaction (PCR) products were purified with a Wizard SV gel and PCR Clean-Up System (Cat# A9281, Promega, Madison, WI, USA) and sequenced on a 3730xl DNA Analyzer (Applied Biosystems, Foster City, CA, USA). In addition, because the variant rs113784532 in *PCDHGA10* is an insertion mutation in a mononucleotide repeat, restriction digestion was used in conjunction with Sanger sequencing to ensure the insertion was not a sequencing artifact. PCR primers 5’-AAGAGTCACCTGATCTTCCC-3’ and 5’-ACACTGG*AG*TAAAAACCAATCTTTT-3’ carried the BpmI restriction enzyme site (underlined sequence), and PCR products were digested with BpmI (Cat# R0565S, NEB) before Sanger sequencing. Nucleotide alterations were confirmed by visual inspection of the electropherograms displayed with Chromas 2.6.5 (Technelysium Pty Ltd, Australia).

### Sanger sequencing of celloidin-embedded human tissue sections

Genomic DNA was extracted from archived celloidin-embedded sections of human temporal bones as described by Wackym et al. with slight modifications [28,29]. Briefly, after rinse of tissue sections in phosphate-buffered saline (PBS) and removal of coverslips, tissues were scraped individually into 1.5ml Eppendorf tubes containing 1:1 volume of ether and 100% ethanol for 2X 30 minutes (min). After centrifugation, the precipitates underwent 3 rounds of washing/centrifugation in double distilled water. The precipitates were then washed twice with 100% ethanol, vacuum dried in a Labconco Centrivap Concentrator (Labconco Corp., Kansas City, MO), and digested with 0.5 mg/ml proteinase K in 500μl of buffer (10 mM Tris, pH 7.5, 50 mM EDTA, 150 mM NaCl, 1% SDS) overnight at 55°C. Genomic DNA was subsequently isolated with standard phenol/chloroform extraction method and used for PCR amplification. Forward and reverse primers for human *PCDHGA10* listed in S1 Table were used to amplify the region containing the variant rs113784532. PCR products were purified and subjected to Sanger sequencing.

### Fluorescent immunostaining

Human temporal bone specimens were previously obtained within 12–24 hours of death from subjects with or without BPPV history [30]. The temporal bones were stored in 10% neutral-buffered formalin at 4°C for 4 weeks, decalcified with 5% EDTA for 9 months, and dehydrated in graded ascending ethylic alcohol and embedded in celloidin over a 3-month period.

The celloidin-embedded temporal bones were cut in 20-micron thick serial sections, and every 10^th^ section was mounted and stained with hematoxylin and eosin. The remaining sections were stored in 80% ethanol until they were used. Sections containing the vestibular ganglia or epithelia were mounted on super frost plus slides (Fisher Scientific) and used for immunofluorescent staining or DNA extraction.

To remove the celloidin [30], sections were placed in a glass Petri dish and immersed in 100% acetone for 2 × 15 min, then sequentially immersed in a mixture of sodium-hydroxide-100% ethanol (1:3) for 10 min; 100% ethanol, 50% ethanol, and distilled water for 5 min each; then rinsed with double distilled water (2x10 min). Slides were placed horizontally in a glass Petri dish containing antigen retrieval solution (Vector Antigen Unmasking Solution, Vector Labs, Burlingame CA diluted 1:250 with distilled water) and heated in the microwave for 5 min, allowed to cool for 15 min, followed by 10 min wash with PBS. A drop of enzymatic antigen retrieval solution 1:5 was added for 3 min (Cat# ab970, Abcam), and sections were washed with PBS 4x15 min.

Sections were blocked in PBS containing 1% normal goat serum (Vector Labs, Burlingame, CA) and 0.1% Triton X-100 (Sigma) for 30 min, and incubated in rabbit-derived polyclonal anti-PCDHGA10 (1:50 in blocking buffer) (catalog # orb1035, Biorbyt, Cambridge, UK) for 48 h at 4°C in a humid chamber. Specificity of this antibody was confirmed by Western blotting as described on the stated supplier’s website. After 3 washes (10 min each) in PBS, Alexa-488 conjugated goat anti-rabbit polyclonal IgG (Molecular Probes, Carlsbad, CA) was added at a dilution of 1:600 and incubated at room temperature for 1 h in the dark. Immunofluorescent background was removed using the Vector True VIEW kit (Vector Labs).

A drop of Vectashield mounting media (Vector Labs, Burlingame, CA) containing DAPI was added to the tissue sections before coverslipping. Digital images were taken using a Zeiss Axio Observer Z1 inverted microscope equipped with an AxioCam MRm camera and with GFP, DsRed and DAPI filter sets.

Negative controls underwent the same procedures, except that non-immune serum was used.

The celloidin-embedded human tissue sections used in the above experiments were approved by the Institutional Review Board (IRB) of UCLA (IRB protocol #10–001449, and 20–000847). All methods used in this study are in accordance with NIH and IRB guidelines and regulations. Appropriate informed consent had been obtained from each patient before inclusion in the study.

### Mice

C57BL/6J (as B6) mice were purchased from the Jackson Laboratory (Bar Harbor, Maine, USA, stock number 000664) and maintained in the vivarium at Boys Town National Research Hospital (BTNRH). All animal procedures were approved by the Institutional Animal Care and Use Committee (IACUC) at BTNRH in accordance with institutional, federal and international guidelines (approval numbers 16–01 and 19–01).

### Fluorescent RNA *in situ* hybridization

#### Generation of digoxigenin-labeled RNA probes

Mouse inner ear tissues were collected, total RNA was purified using the RNeasy Plus Mini kit (Cat# 74134, Qiagen Inc., Valencia, CA, USA) combined with extra on-column DNase treatment with an RNase-free DNase set (Qiagen), and first-strand cDNA was generated using SuperScript^®^ VILO^™^ MasterMix (Invitrogen, Grand Island, NY) according to the manufacturers’ instructions. The resultant product was used as PCR template to amplify the cDNA sequences for generating RNA probes for *in situ* hybridization. Primers 5’-ACACTCGAGCTGTGAGAAAAAAGATCC-3’ and 5’- ACATCTAGAGAAACGCCAGTCAGTG-3’ were used to amplify a 109bp sequence for detecting mouse *Pcdhga10* long isoform, and primers 5’-ACACTCGAGCTGTGAGAAAAAAGATCC-3’ and 5’-ACATCTAGATTTGGGCTCAAGCACAACG-3’ were used to amplify a 143bp sequence for detecting a mouse *Pcdhga10* short isoform that is at the equivalent base position of human *PCDHGA10*.

Amplification products were purified using QIAquick PCR purification kit (Qiagen) and cloned into pGEM-T easy vector (Promega, Madison, WI). X-Gal/IPTG-selected clones were digested with restriction enzymes to confirm the size of inserts, then sequenced for further confirmation as well as determination of the orientation. The confirmed clones were linearized with *XhoI* or *XbaI*, and used to generate digoxigenin‐labeled single‐strand antisense and sense RNA probes for hybridization using the DIG RNA Labeling Kit (SP6/T7) (Cat# 11175025910, Roche Molecular Biochemicals, Alameda, CA) according to the manufacturer’s instructions.

#### Tissue preparation

Animals were deeply anesthetized with ketamine-xylazine (ketamine: 200 mg/kg; xylazine: 5 mg/kg body weight) and then decapitated. All following steps were carried out under RNase-free conditions with diethyl pyrocarbonate (DEPC)-treated solutions or buffers made with DEPC-treated water. Inner ears were dissected in PBS and fixed in 4% paraformaldehyde (PFA) in PBS for 4 hours at room temperature, decalcified in 0.25 M EDTA (pH7.4) overnight, dehydrated in 30% sucrose prepared in PBS, embedded in optimal cutting temperature (O.C.T) compound at below -20°C, sectioned at 9 μm using a MICROM HM-505 N cryostat (Microm, Germany), thaw-mounted on Superfrost Plus glass slides (Thermo Fisher Scientific) and air-dried. The sections were stored at -80°C until processed for fluorescent in situ hybridization (FISH).

#### RNA in situ hybridization

Frozen tissue sections were warmed to room temperature and desiccated for 20 min at 50°C, post-fixed in 4% PFA, followed by two washes in PBS. Then sections were treated with 5 μg/ml proteinase K (Thermo Fisher Scientific) in 50 mM Tris buffer, pH 8.0, containing 5 mM EDTA, for 5 min at 37°C. After two washes in 2X saline sodium citrate (SSC), sections were acetylated with 0.1 M triethanolamine, pH 8.0, containing 0.25% (v/v %) acetic anhydride for 20 min, and rinsed in 2X SSC. Sections were treated with pre-warmed pre-hybridization solution composed of 50% formamide, 4X SSC, 10% Dextran sulfate, 1X Dernhardt’s and 50 μg/ml yeast tRNA (Sigma-Aldrich, St. Louis, MO, USA) in an RNase-free humid chamber containing a thin layer of 3M Whatman paper soaked with 4X SSC/50% formamide for 2 h at 55°C. The pre-hybridization solution was then replaced with pre-warmed hybridization buffer containing 0.3 mg/ml sheared, denatured herring sperm DNA and 0.5 μg/ml digoxigenin-labeled anti-sense/sense RNA probe, and the tissue sections with cover slips were incubated overnight at 55°C in the humid chamber.

Following hybridization, sections were rinsed in 2X SSC to wash-off the cover slips and treated with 20 μg/ml RNase A (Sigma-Aldrich) in 10 mM Tris, pH 8.0, containing 500 mM NaCl and 1 mM EDTA, for 30 min at 37°C, followed by additional stringent washes in 2X SSC/50% formamide (5 min), 1X SSC (5 min), 0.5X SSC (5 min), at 50°C and three washes in PBS at room temperature. After that, sections were stained using the Alexa Fluor^™^ 488 Tyramide SuperBoost^™^ Kit (Cat# B40922, Thermo Fisher Scientific) combined with rabbit-derived monoclonal digoxigenin antibody (Cat# 700772, Thermo Fisher Scientific) according to the manufactures instructions. Briefly, sections were blocked with 10% normal goat serum for 60 min at room temperature, and incubated with digoxigenin antibody (1:500 in blocking buffer) overnight at 4°C, followed by three washes in PBS. Sections were then incubated with poly-HRP-conjugated goat anti-rabbit secondary antibody (1: 600 in blocking buffer), together with DAPI (Sigma-Aldrich, St. Louis, MO, USA) at a dilution of 1:10,000, in the dark for 60 min at room temperature. After three washes in PBS, for the signal enhancement, the tyramide working solution was applied to the sections for 6 min at room temperature, and the stop solution was used to terminate the HRP reaction. After three more washes in PBS, sections were mounted in Fluoromount-G and fluorescence images were acquired using a Zeiss Axio Observer Z1 inverted microscope equipped with an AxioCam MRm camera and with GFP, DsRed and DAPI filter sets.

### Statistical analysis

Statistical significance of the difference in allele frequencies among BPPV families, non-BPPV families or the general population in public genome/exome databases was evaluated by Fisher’s exact test (two-tailed). Relatedness across families with exome data were calculated using the KING software described by Manichaikul et al. [31]. Age differences in the affected samples with and without the identified variant were compared by two-tailed Student’s t-test. RNA expression levels among different age groups were compared with one way ANOVA with Bonferroni correction.

## Results

### Characteristics of families

**Fig 1:**
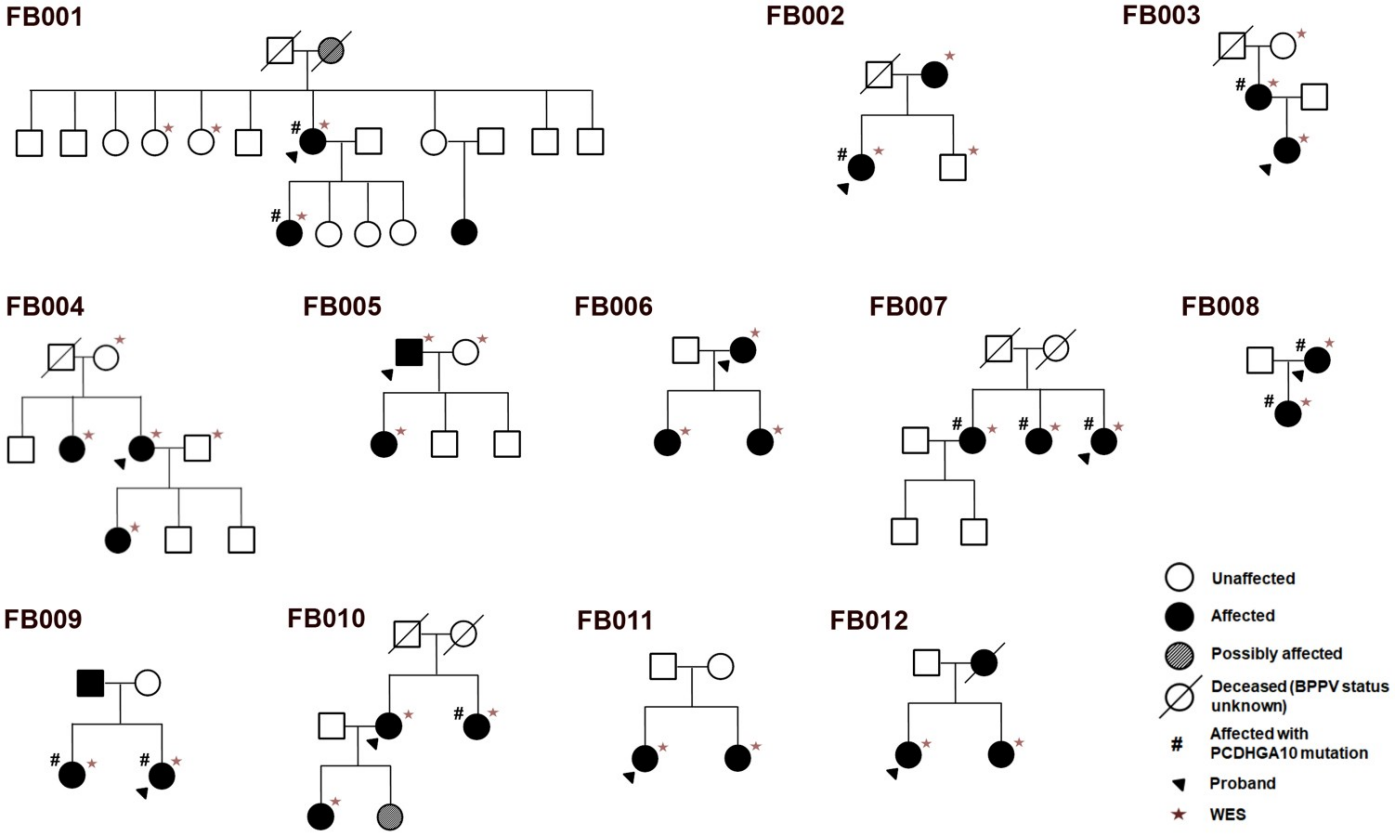
Pedigrees of the 12 BPPV families selected for whole exome sequencing (WES) including UTR. Filled arrowheads indicate probands. Circle and square symbols represent female and male individuals, respectively. Symbols with slashes indicate deceased individuals. The BPPV status of deceased individuals is unknown unless the circle or square is shaded or filled. Affected and unaffected family members are denoted in filled and unfilled symbols, respectively. Asterisks indicate the family members selected for WES, and # indicates those carrying the PCDHGA10 variant rs113784532. FB: Familial BPPV.

WES (+UTR) analysis was performed on 12 BPPV families of non-Hispanic white from the US Midwest, each family with 2–3 affected individuals and 0–2 of unaffected blood relatives. For the few families with no unaffected samples at the time, independent controls were used in the analysis (see Method section for selection criteria). In total, WES was performed on 28 affected individuals, 7 unaffected relatives and 7 independent controls (Fig 1). Individuals denoted with asterisks in the figure were sequenced.

Families in this study all had recurrent BPPV. BPPV has a reported mean onset age of 49.4 years [5]. In our WES samples that had definitive information on onset ages, the mean onset age was 46.1±13.1 (n = 21), and the mean age of unaffected subjects was 66.4±10.0 (n = 14). S3 Table shows the ages and BPPV-onset ages of the individuals. Our sample sets had a female/male ratio of 2.1:1, which is consistent with previous reports of higher prevalence of BPPV in females [5,18,32].

### Whole exome sequencing and mutation detection

**Fig 2:**
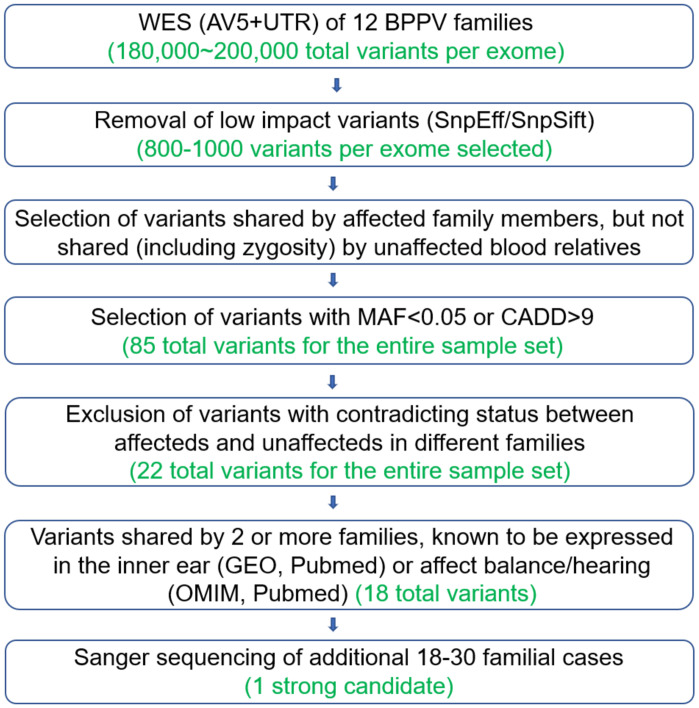
Tiered selection of variants from whole exome sequencing of 12 BPPV families.

Each exome had at least 97.6% covered at >10x, and 93.0% at >20x, with an average coverage of 63-fold. Approximately 180,000–200,000 single nucleotide variants (SNVs) and insertion-deletions (indels) per exome were detected. After variants were filtered as described in Methods, a total of 800–1000 variants per exome remained. A tiered approach was used for variant selection, as depicted in Fig 2 and described in Methods. Since we anticipated genetic heterogeneity, we first considered each family separately and selected the variants (including differences in homozygous and heterozygous state) that were shared among all affected individuals in the family but not among unaffected members. This resulted in 13–227 variants per family. Next, we selected variants with MAF<0.05 or CADD>9, which narrowed down the list to a total of 85 variants for the entire sample set. By comparing all families together, we excluded variants with contradicting status among affected and unaffected subjects in different families, resulting in a total of 22 selected variants from all families. Finally, variants that were shared by 2 or more families, or were within genes known to be expressed in the inner ear according to GEO and Pubmed, or known to affect balance/hearing according to OMIM and Pubmed, were given higher priority for follow-up. A total of 18 variants (S1 Table) remained at this stage. Sanger sequencing of the 18 variants in additional 18–30 BPPV families (2–3 individuals per family) resulted in an exceedingly strong candidate variant (Table 1 and S2 Table). Sanger sequencing of the 18 variants was also performed on the WES samples, which confirmed the WES results. These 18 variants are located in the following 17 genes: *PCDHGA10*, *CASP10*, *TMEM119*, *NOD2*, *STARD6*, *MYBPC3*, *MPO*, *BAG3*, *CP*, *LRP2*, *SYNE2*, *LMNB2*, *DMD*, *GPR98*, *CIDEC*, *RYR2*, *ANO10*.

**Table 1:** Variants observed in BPPV versus non-BPPV families.

Gene	Chr	ref	alt	dbSNP	Mutant allele count (+/-)			
					BPPV families	Non-BPPV families	ExAC	NHLBIEA
*PCDHGA10* (P-values)	chr5		A	rs113784532	13/29	1/49 (1.80x10^-4^)	11/2739 (5.85x10^-19^)	476/3054 (4.90x10^-3^)

A total of 42 BPPV families and 50 non-BPPV families were analyzed for rs113784532, and one proband from each family was used for allele frequency calculations. Data are presented as number of families positive (+) for the variant over those negative (-) for it. NHLBI exome sequencing data of European Americans are shown here. Chr, chromosome; EA, European Americans.

In all, a total of 42 BPPV families and 50 non-BPPV families (also 2–3 individuals per family) were analyzed, and one proband from each family was used for allele frequency calculations.

**Fig 3:**
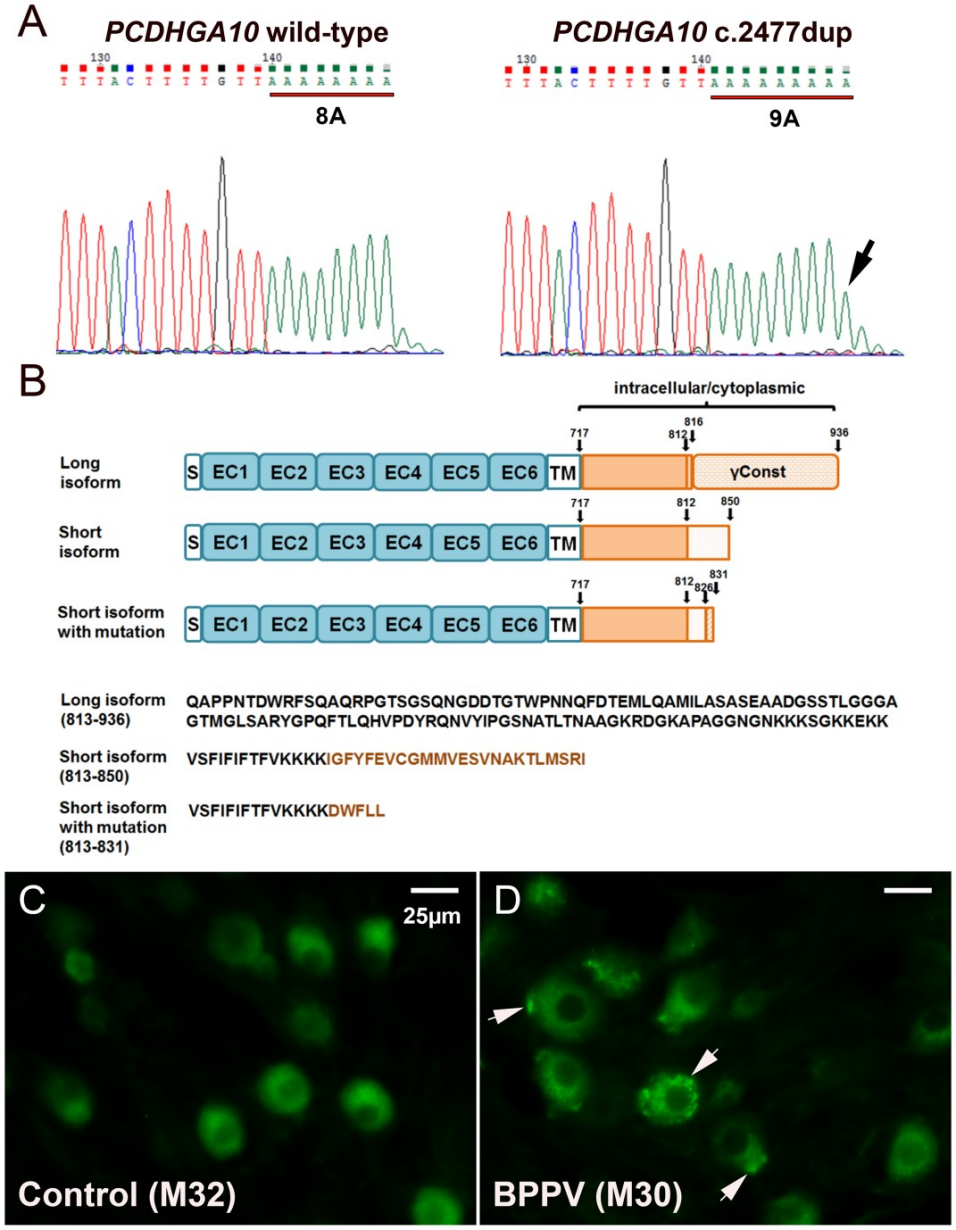
Frameshift variant causes premature stop, truncating the PCDHGA10 short isoform. A, Verification of the NM_032090.1:c.2476_2477dup in *PCDHGA10* by Sanger sequencing after BpmI restriction digestion. B, Protein structures of PCDHGA10 long, short and mutant isoforms. EC, extracellular; TM, transmembrane; Const: Constant domain. C, Fluorescent immunostaining shows that wildtype PCDHGA10 has no large aggregates in the cytosol of non-BPPV samples at young ages (the vestibular ganglia is shown). D, PCDHGA10 with the rs113784532 mutation forms large intracellular aggregates in BPPV samples even at young ages. Arrows indicate aggregates. M30 and M32, male at 30 and 32 years of age, respectively. Scale bar, 25μm.

The strongest candidate is a heterozygous insertion variant rs113784532 (NM_032090.1: c.2477dup, previously known as rs369101565/rs752029921/rs750612188), in the gene protocadherin gamma A10 (*PCDHGA10*) (Fig 3A). Control subjects all had 11 As (8 after BpmI restriction digestion as shown in the figure), whereas many BPPV subjects had 12 As (9 after digestion). This frameshift variant causes a premature stop, truncating the PCDHGA10 short isoform (Fig 3B). The affected region of the short isoform is normally expressed from a segment of the intron in the long isoform [33], and the wildtype form of this segment is expressed in the RIKEN cerebellum cDNA library as well as in the inner ear (see data in the section below). PCDHGA10 with the rs113784532 mutation formed large intracellular aggregates in BPPV samples even at young ages (Fig 3D). In contrast, wildtype PCDHGA10 protein showed no such aggregates in the cytosol of non-BPPV samples (Fig 3C).

In our sample set, 13 out of 42 BPPV families had this variant (Table 1, p = 5.85x10^-19^ vs. ExAC data on all populations; p = 4.90x10^-3^ vs. NHLBI exome data on European Americans). Individuals carrying this variant are labeled with # in Fig 1. This variant also tends to make the onset age of recurrent BPPV earlier, with an average onset age of 44.0±14.0 (n = 16, only those with definitive information on onset ages were tallied) years old among those with the variant as compared with 54.4±16.1 (n = 36 tallied) years of onset age among those without (p = 0.054, S3 Table).

One non-BPPV family was positive for the rs113784532 mutation (Table 1). Currently, it is not clear whether it was due to some kind of functional compensation by another unidentified gene, although BPPV is known to have reduced penetrance (see Introduction section for more information). Our current data show that the penetrance is approximately 95%. Sanger sequencing of the entire coding region of *PCDHGA10* in our sample set did not identify any additional likely causative variants.

Analysis of relatedness statistic using the KING software showed that only within families were there kinship coefficients greater than 0.125, suggesting no relationships closer than 3^rd^ degree between families studied.

### Pcdhga10 expression in the mouse vestibule

**Fig 4:**
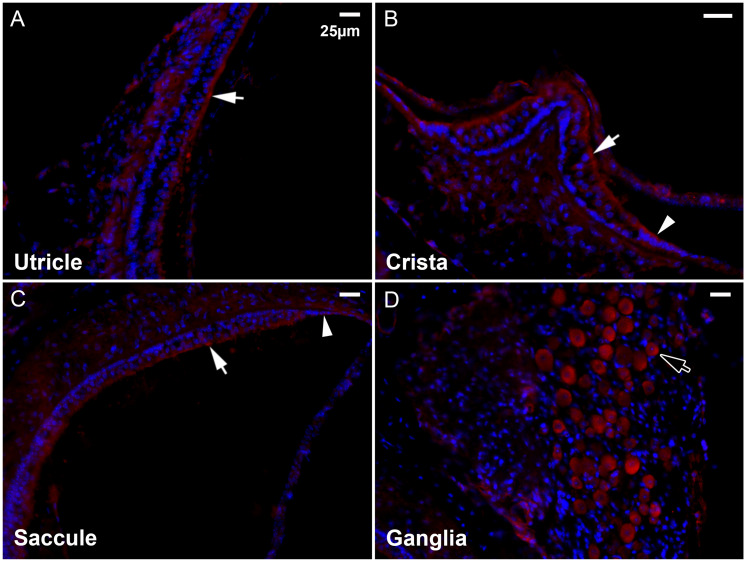
Fluorescent immunostaining of Pcdhga10 in the murine vestibule at 2 months of age. Arrows indicate hair cells, arrowheads transitional epithelial cells and hollow arrow vestibular ganglia. Scale bar, 25μm.

Fluorescent immunostaining was carried out to examine the expression of PCDHGA10 in the mouse inner ear. The highest level of expression was observed in the ganglia of the vestibule (Fig 4D) and cochlea (not shown), in both types of ganglia cells. There was a low level of expression in vestibular transitional epithelia (arrowheads in Fig 4B and 4C) and hair cells (arrows in Fig 4A–4C).

**Fig 5:**
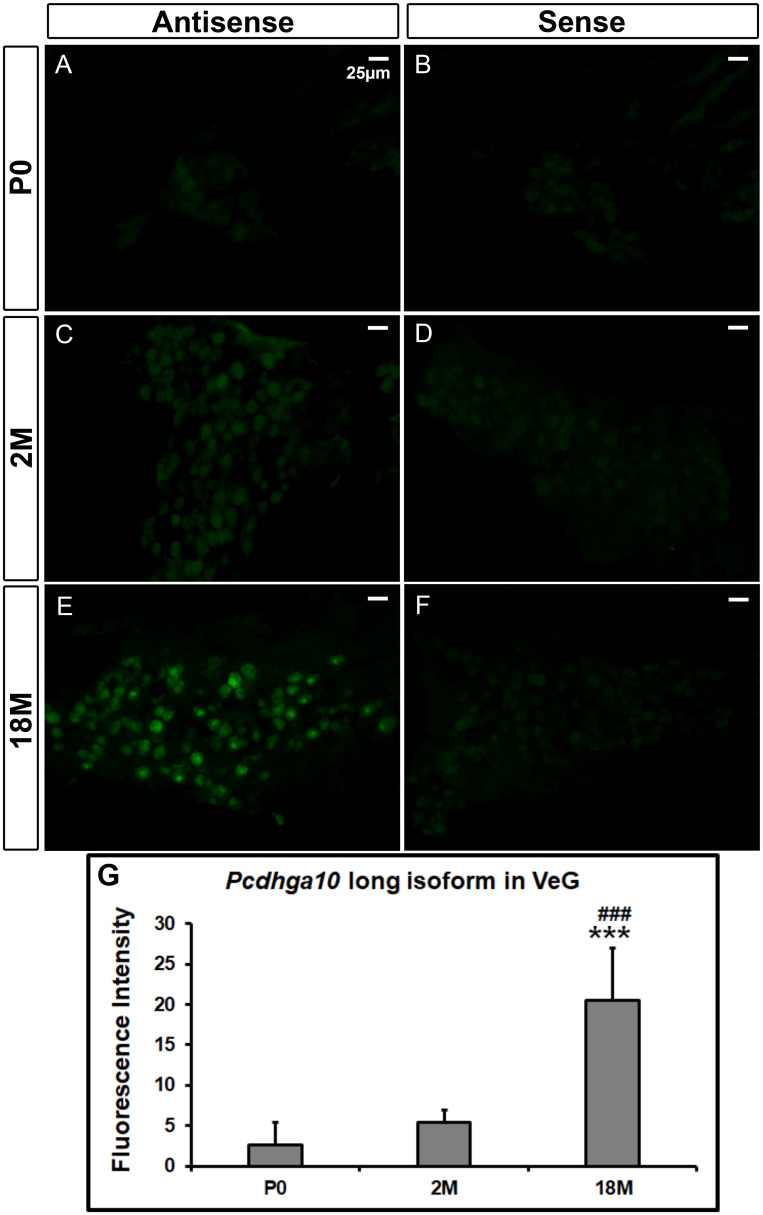
Fluorescent *in situ* hybridization of the long isoform of *Pcdhga10* mRNA in mouse vestibular ganglia. At P0 (A) and 2M (C), the fluorescent signals of the antisense probe of the long isoform are absent (or similar to that in the negative controls at the same ages) (B & D), whereas at 18M (E), the fluorescent signal of the antisense probe is much stronger than the negative control (F). P0, postnatal day 0; 2M and 18M, 2 and 18 months old, respectively. VeG, vestibular ganglia. Scale bar, 25μm. *** and ### denote p<0.001 when the 18M group is compared with P0 and 2M, respectively (n = 4 mice/group).

**Fig 6:**
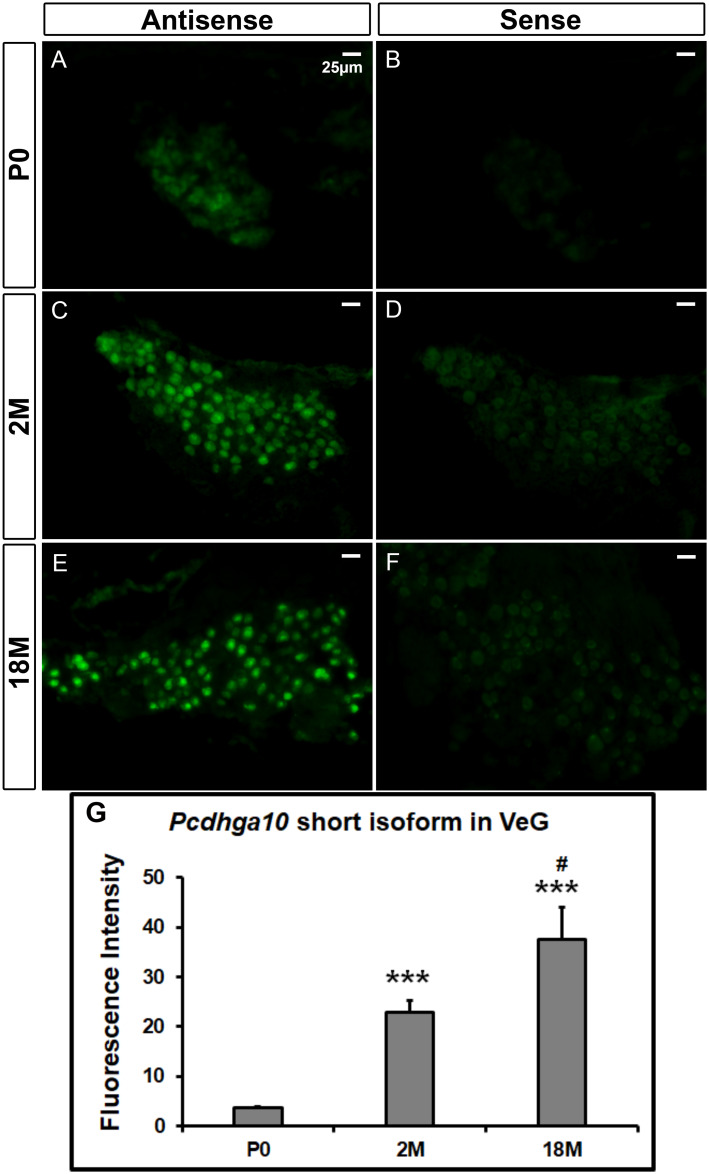
Fluorescent *in situ* hybridization of the short isoform of *Pcdhga10* mRNA in mouse vestibular ganglia. Fluorescent signals of the antisense probe of the short isoform increase with age, whereas signals of the negative controls are at background levels. P0, postnatal day 0; 2M and 18M, 2 and 18 months old, respectively. VeG, vestibular ganglia. Scale bar, 25μm. *** denotes p<0.001 when compared with P0, # denotes p<0.05 when the 2M group is compared with 18M (n = 4 mice/group).

We then examined the distribution of the long and short isoforms of mouse *Pcdhga10* mRNA at different ages using sensitive fluorescent *in situ* hybridization (FISH). No existing mice (wildtype or mutant) carry the rs113784532 mutation, so wildtype inbred mice were used for the expression studies. FISH confirmed that *Pcdhga10* was more highly expressed in the ganglia of the inner ear. In the vestibular ganglia of mice at postnatal day 0 (P0) and 2 months old (2M), expression of the long isoform was absent or extremely low (Fig 5A and 5C), whereas at 18 months of age (18M, Fig 5E), expression was moderate (ANOVA *F*_*(2*,*15)*_ = 29.28, *P* = 6.62x10^-6^, Bonferroni correction *P* = 6.78x10^-4^ vs. age P0 and *P* = 4.97x10^-4^ vs. age 2M). In contrast, the expression of *mPcdhga10* short isoform was already detectable, although not very high, at P0 (Fig 6A), and the fluorescent signals were even stronger in the vestibular ganglia of 2M and 18M mice (Fig 6C and 6E) (ANOVA *F*_*(2*,*16)*_ = 37.52, *P* = 9.10x10^-7^, Bonferroni correction *P* = 4.23x10^-5^ vs. age 2M, *P* = 2.64x10^-5^ vs. age 18M and *P* = 0.02 when 2M was compared with 18M). All the *in situ* hybridizations had controls using the corresponding sense probes on adjacent sections under the same experimental conditions. No hybridization signals were seen in controls, indicating that no nonspecific binding to RNA or DNA occurred. The data demonstrate that the expression level of *Pcdhga10* short isoform was much higher than that of the long isoform at all age groups, suggesting that the short isoform is the predominant *Pcdhga10* in the tissue.

## Discussion

To our knowledge, this is the first study to identify genetic mutations causing or exacerbating idiopathic recurrent BPPV in humans (other than the intronic variant recently identified by Deng et al. [22] through sequencing 3 candidate genes). Given the high prevalence and debilitating nature of recurrent BPPV, this type of genetic studies is overdue.

Our data show that a variant in the short isoform of the *PCDHGA10* gene is strongly associated with familial cases of recurrent BPPV. This variant alone can account for at least 31% of the familial cases of BPPV we studied (a total of 42 families studied). In some families, not all affected individuals carry the identified PCDHGA10 variant (see Fig 1). Some family members were unwilling to donate their DNA samples, or unable to make such a decision, making it difficult to assess co-segregation of rs113784532 and BPPV in a few families and determine whether a few individuals (e.g. the proband’s sister who has an affected daughter in FB001) are obligatory carriers. It is highly likely that there are other genetic, epigenetic or non-genetic causes, which would be interesting for future studies.

PCDHGA10 belongs to the γ-subfamily of the protocadherins (PCDH) super family of 70 genes. PCDHs are neuronal adhesion molecules that are concentrated in neurons and synapses. Fifty-eight of the PCDHs are tandemly arrayed in three clusters (α, β, and γ) on human chromosome 5 and mouse chromosome 18. Each cluster encodes a constant domain and multiple variable domains including the extracellular, transmembrane, and cytoplasmic domains of individual isoforms. The constant domain of PCDHs is identical within the same cluster, and highly conserved between the three clusters. Within the Pcdh-α and γ clusters, each variable exon is transcribed from its own promoter and spliced to constant exons that encode a shared C-terminal constant domain [34–37], much like immunoglobulin (Ig) and T cell receptor (TCR) gene clusters. Such diversity and genomic rearrangement, coupled with evidence that different neurons express different PCDHs [36,38–43], suggest that defined sets of PCDH expression may engage in the establishment of specific neuronal connections.

Unlike classical cadherins, which are present at the cell surface, γ-Pcdhs have a prominent intracellular presence in neurons, particularly in endolysosomes [40,41,44]. The variable cytoplasmic domain (VCD) of γ-PCDHs is the most important contributor to localizing the protein to endolysosomes [45,46]. When the VCD of γ-PCDHs is truncated, the protein is no longer distributed in endosomes/lysosomes (as it normally is) and organelle trafficking is abnormal in cell culture [45]. This is because γ-PCDHs induce tubulation of organelles in the late endosome/lysosome pathway, which is a rate-limiting factor in trafficking of the organelles. The VCD segment (near our variant rs113784532) of γ-PCDHs is responsible and necessary for such function, and for targeting the protein to the organelles and their trafficking.

Structural homology analysis using Phyre2 [47] shows with 96.8% confidence that the 40 amino acids in the C-terminal region (except the last 4 residues) of PCDHGA10 short isoform adopt the conformation of the Extracellular Cadherin 1 (EC1) domain of mouse Pcdhgb14 and Pcdha4 (PDB accession c1wyjA_ and c1wuzA_, respectively). The frameshift mutation rs113784532 in our samples truncates the C-terminal 24 amino acids in this domain, leading to absence of this protein fold. We postulate that this domain in wildtype PCDHGA10 may participate in targeting the protein to autophagosomes-lysosomes for degradation, and the mutation renders the cellular disposal machinery unable to recognize and remove the mutant protein. Our fluorescent immunostaining results show that mutant PCDHGA10 forms large intracellular aggregates in BPPV samples even at young ages (Fig 3). The conceivable consequence would be deficits in the function of the cells expressing the mutant protein. Alternatively, the truncation may lead to nonsense-mediated decay (NMD) of the transcript, although evidence in the literature shows that the mRNA surveillance pathway that causes NMD is inefficient when the premature termination codon mutations are located in the last exon [48–50]. The PCDHGA10 variant we detected is located at the 3’ end of the short isoform, therefore, it will likely escape the surveillance of NMD, producing a truncated protein which forms aggregates as shown in Fig 3.

The vestibular transitional epithelia and hair cells, where there is a low level of Pcdhga10 expression (Fig 4), normally produce many of the proteins critical for otoconia formation and maintenance [51]. These cells would likely be affected by the mutant PCDHGA10 and reduce the production of otoconial proteins, leading to compromised otoconia formation and/or maintenance. Currently, it is unknown whether some cases of the degenerative otoconia observed in the elderly [52–54] are caused by this mutation, but our data clearly show earlier onset of BPPV in the subjects with this mutation.

More studies are needed to see if there is a neuronal contribution to the etiology of some cases of recurrent BPPV, given the expression of *Pcdhga10* in vestibular ganglia. Such as scenario may explain why vestibular maneuvers (i.e. the Epley and Semont maneuvers), designed to move the dislocated otoconia back into the utricle, do not work well or only work for a short period of time for some BPPV patients. Indeed, there is a 50% ganglia loss [55] observed in the postmortem vestibules of BPPV patients. In fish, vestibular neuroectomy causes abnormal otoliths (otoconia are called otoliths in fish) [56]. Therefore, the ganglia loss may worsen otoconia degeneration, which would facilitate its dislocation into the canals. The ganglia deficits or loss may also cause imbalanced neurotransmission, loss of contralateral inhibition, or as Gacek postulated [55], loss of inhibition by the otolithic organ on the crista in the canals.

Further studies are also needed to confirm the biological consequences of the potential candidate variants, as well as identify additional genetic mutations, including variants in gene regulatory regions, that may contribute to the etiology of BPPV. Given the female predominance in BPPV cases (including those with rs113784532), it would be interesting to examine hormonal (i.e. estrogen) influence on the expression, trafficking and function of PCDHGA10.

Among the strengths of the study are the family-based case-control approach and the large number of families studied, which compensated for the non-classical Mendelian characteristic of the phenotype. The caveat is that the family-based approach can complicate the analysis due to potential dependence of individuals between some families. However, kinship analysis using exome data showed no relationships closer than 3^rd^ degree among the families studied. Because the families used in exome sequencing were random in terms of family structure and kinships across families, we assumed that the additional families which only underwent Sanger sequencing were not related either.

## Conclusions

Our data show that a variant in the *PCDHGA10* gene may underlie some familial cases of recurrent BPPV. The mutant protein forms large aggregates in BPPV samples even at young ages. Thus, our study provides some insight into the etiology of idiopathic recurrent BPPV. These findings can be of immediate benefit to the families from several aspects. Firstly, the information can be used for diagnosis and counseling. Results from genetic testing can provide mental relief for the affected families because they feel that at least they know what is causing their intense vertigo. Secondly, there may be generic remedies available already. For example, if the gene mutation causes protein aggregation, remedies to stimulate autophagy are already available and tested in animals and cell culture. Thirdly, depending on the severity and type of variant involved in causing recurrent BPPV, gene therapies such as using RNAi and oligos, or genome editing using CRISPR/Cas9 can be implemented on recurrent BPPV patients in the future.

## Supporting information

S1 Table Variants and primers used in Sanger sequencing. The variants were selected from whole exome sequencing.
(DOCX)

S2 Table Results of Sanger sequencing of the other 17 variants in BPPV families as compared with public databases. (DOCX)

S3 Table Ages and BPPV-onset ages of samples. (XLSX)
