# Supplementary material for: Identification of a genetic variant underlying familial cases of recurrent benign paroxysmal positional vertigo
Source: PLoS One. 2021 May 6;16(5):e0251386. doi: 10.1371/journal.pone.0251386 (PMC8101739; doi:10.1371/journal.pone.0251386)
Supplement: S1 Table — The variants were selected from whole exome sequencing. (DOCX) [file pone.0251386.s001.docx]

**S1 Table.** Variants and primers used in Sanger sequencing

| **Gene** | **dbSNP** | **Primer sequence (5’>3’)** | | **Annealing Temperature for PCR (°C)** |
| --- | --- | --- | --- | --- |
|  |  | **Forward** | **Reverse** |  |
| *PCDHGA10* | rs113784532 | AAGAGTCACCTGATCTTCCC | ACACTGGAGTAAAAACCAATCTTTT | 55 |
| *CASP10* | rs13010627 | AGGCCCTCATTCCCATTCG | TATACCAGCTGCCTTCCTC | 55 |
| *TMEM119* | rs144109867 | CTGCTGATGTTCATCGTCTG | GCTGCCCTTCTCCTCTTC | 60 |
| *NOD2* | rs2066847 | TCTTCTTTTCCAGGTTGTCC | GCCTTACCAGACTTCCAG | 55 |
| *STARD6* | rs17292725 | AAACTTGCAGATAGATGGAC | TCTTACTGAAGTTTTAACCAC | 55 |
| *MYBPC3* | rs3729986 | CTTTGCTCACAGGGTCAAG | ACAGCAGCTCACACTCAC | 55 |
| *MPO* | rs119468010 | GCATTGACCCCATCCTCC | TGCGCTGCATGTTCAGAG | 60 |
| *BAG3* | rs35434411 | CTCCATCCTCTGCCAATG | TCAGTTCGGAATCGCTGC | 60 |
| *CP* | rs61733458 | GCACCCACAGAAACATTC | AGTTGGACTTACCTGTCTC | 55 |
| *LRP2* | rs17848169 | TAGGCAGAGGATTGAACGC | TGGTGCACAGATATGGCTG | 60 |
| *LRP2* | rs34291900 | TTCCTTCCAGCTACCAATC | GCGCTCATCTGTATCCAG | 55 |
| *SYNE2* | rs12881815 | ACATTGGTGGAAAACACGC | AAAGGCTGACCTGAGCAG | 55 |
| *LMNB2* | rs121912497 | CATTTTTGAGCCCTCCCTTG | CTTGGCCTGGTAGGTCTGC | 55 |
| *DMD* | rs72468681 | TTTTTCTTTCTAGAGGGTG | TTGCTGTTGGCTCTGATG | 55 |
| *GPR98* | rs111033530 | TTAGGAGTTCCACCAGCC | GAATTGTTATGTTAGCCTCTTG | 55 |
| *CIDEC* | rs61742367 | CCAGGCATGTGTCAGTGC | GGTCCTCAAGACTGTAAGC | 55 |
| *RYR2* | rs56229512 | TGCTTATTGTTAGTCCTCTG | AAGCTGCAACCTCATACC | 55 |
| *ANO10* | rs17409162 | TTTCTTTTAGTTGGCTTTTG | CTTTACTTACCTCCACTG | 52 |
