# Supplementary material for: Identification of a genetic variant underlying familial cases of recurrent benign paroxysmal positional vertigo
Source: PLoS One. 2021 May 6;16(5):e0251386. doi: 10.1371/journal.pone.0251386 (PMC8101739; doi:10.1371/journal.pone.0251386)
Supplement: S2 Table — (DOCX) [file pone.0251386.s002.docx]

**S2 Table.** Other variants observed in BPPV families versus public data [1000 Genome (1000G) and NHLBI data on European Americans (EA)].

| **Gene** | **Chr** | **ref** | **alt** | **dbSNP** | **Mutant allele count (+/-)** | | |
| --- | --- | --- | --- | --- | --- | --- | --- |
|  |  |  |  |  | **BPPV families** | **1000G** | **NHLBIEA** |
| *CASP10*  (P-values) | chr5 | G | A | Rs13010627 | 8/29 | 88/2412  (4.89x10^-5^) | 527/3753  (0.13) |
| *TMEM119* (P-values) | chr12 | G | A | rs144109867 | 1/29 | 1/2503  (0.02) | 9/4280  (0.07) |
| *NOD2*  (P-values) | chr16 |  | C | rs2066847 | 2/28 | 30/247  (0.05) | 181/3946  (0.38) |
| *STARD6*  (P-values) | chr18 | G | A | rs17292725 | 2/28 | 71/2433  (0.21) | 334/3966  (1.00) |
| *MYBPC3*  (P-values) | chr11 | C | T | rs3729986 | 3/27 | 161/2343  (0.44) | 694/3462  (0.46) |
| *MPO*  (P-values) | chr17 | G | A | rs119468010 | 2/28 | 2/2502  (8.00x10^-4^) | 25/4275  (0.01) |
| *BAG3*  (P-values) | chr10 | G | A | rs35434411 | 5/34 | 37/2467  (4.00x10^-4^) | 266/4027  (0.09) |
| *CP*  (P-values) | chr3 | G | A | rs61733458 | 4/39 | 98/2402  (0.09) | 257/4039  (0.33) |
| *LRP2*  (P-values) | chr2 | T | C | rs17848169 | 1/29 | 86/2419  (1.00) | 318/3982  (0.72) |
| *LRP2*  (P-values) | chr2 | C | T | rs34291900 | 2/28 | 72/2432  (0.22) | 298/4002  (0.16) |
| *SYNE2*  (P-values) | chr14 | G | A | rs12881815 | 2/28 | 93/2411  (0.31) | 423/3877  (0.76) |
| *LMNB2*  (P-values) | chr19 | C | T | rs121912497 | 2/40 | 23/2481  (0.06) | 116/4170  (0.32) |
| *DMD*  (P-values) | chrX | A | C | rs72468681 | 1/29 | 11/2493  (0.13) | N/A |
| *GPR98*  (P-values) | chr5 | G | A | rs111033530 | 2/28 | 24/2480  (0.04) | 133/4021  (0.25) |
| *CIDEC*  (P-values) | chr3 | G | A | rs61742367 | 1/29 | 65/2439  (0.55) | 256/3966  (1.00) |
| *RYR2*  (P-values) | chr1 | A | G | rs56229512 | 1/29 | 36/2468  (0.36) | 178/3948  (1.00) |
| *ANO10*  (P-values) | chr3 | G | A | rs17409162 | 2/28 | 177/2327  (1.00) | 544/3756  (0.58) |
